# ﻿Re-evaluating monotypic *Eleutherostylis* from New Guinea and the Moluccas and its inclusion in *Grewia* (Malvaceae, Grewioideae)

**DOI:** 10.3897/phytokeys.237.114105

**Published:** 2024-01-19

**Authors:** Laurence J. Dorr, Kenneth J. Wurdack

**Affiliations:** 1 National Museum of Natural History, Department of Botany, MRC-166, Smithsonian Institution, P.O. Box 37012, Washington, D.C. 20013-7012, USA Smithsonian Institution Washington D.C. United States of America

**Keywords:** *Eleutherostylis*, *Grewia*, lectotype, Malvaceae, neotype, phylogeny

## Abstract

Morphological and molecular phylogenetic evidence indicate that *Eleutherostylis* Burret (Malvaceae, Grewioideae), a monotypic genus described from New Guinea, is best considered a synonym of *Grewia* L., a species-rich genus widespread across the Paleotropics and found in Africa, Arabia, Asia, Australia and the western Pacific. A new combination, based on *E.renistipulata* Burret, *G.renistipulata* (Burret) Dorr, **comb. nov.**, is proposed. Original material of the basionym could not be located and a neotype is designated. A lectotype is designated for *G.morotaiensis* Kosterm., a synonym of *G.renistipulata*.

## ﻿Introduction

Generic circumscriptions in Malvaceae s.l. remain surprisingly unsettled and many genera of clearly uncertain circumscription have not had their monophyly tested in well-sampled phylogenies. Sufficient taxon sampling in such phylogenies, even with limited genetic loci, can often identify problems and suggest alternative taxonomies (e.g. *Eriolaena* DC., see Dorr and Wurdack (2021)). *Grewia* L. (Malvaceae, Grewioideae), one of the largest genera in the family, with 280–300 species distributed across Africa, Arabia, Asia, Australia and the western Pacific has a history of generic circumscription problems with multiple segregates. One of those segregates, the monotypic genus *Eleutherostylis* Burret, endemic to New Guinea and the Moluccas, is the focus of this study. When Burret (1926) created *Eleutherostylis*, he stated how it differed from *Grewia* while tacitly admitting the two genera were closely allied. He observed that his new genus differed by having several styles that are free to the base and a gynoecium with more locules. *Eleutherostylis* has 4(5) locules while *Grewia* sensu Burret has 1–2(3) locules. These two genera along with *Vincentia* Bojer (non Gaud.) and *Microcos* L. were the only genera Burret (1926) included in his circumscription of Grewieae Endl. (Grewioideae).

Hutchinson (1967) retained *Eleutherostylis* in an expanded circumscription of Grewieae in which he included seven genera. His concept of the genus *Grewia* included *Vincentia* Bojer and *Microcos* as synonyms rather than distinct genera. In Hutchinson’s (1967) key to the tribe, *Eleutherostylis* was distinguished from *Grewia* by a single character, viz. flowers dioecious versus flowers bisexual. Earlier, Burret (1926) had rather pointedly dismissed floral sexuality as a useful systematic character (“Systematischer Wert ist [die Geschlechtsverh] nicht beizumessen”). Hutchinson also noted that he did not follow Burret (1926) who retained *Vincentia* Bojer and *Microcos* as genera separate from *Grewia* because the “stigmatic characters used for distinguishing [these two genera] are scarcely of generic importance”. The former is considered now a synonym of *Grewia* (Capuron and Mabberley 1999) while there is strong evidence the latter is distinct (see Brunken and Muellner (2012)).

Few other authors have discussed the relationships of *Eleutherostylis*. Kostermans (1972) considered the genus to be close to both *Grewia* and *Trichospermum* Blume and he placed *G.morotaiensis* Kosterm., described from the Moluccas, in synonymy under *E.renistipulata*. Bayer and Kubitzki (2003) maintained *Eleutherostylis* as a distinct genus, separating it from *Grewia* by the lack of a style in female flowers (versus styles cylindrical or dilated apically) and its heteromorphic (versus usually monomorphic) stipules. The latter is scarcely a generic character and positionally varies within a plant (heteromorphic pairs of reniform plus filiform stipules on lateral plagiotropic branches, but only reniform stipules on orthotropic branches). Brunken and Muellner (2012) included *Eleutherostylis* in their circumscription of the Grewieae and within that tribe in their “Grewia subclade”. They based their argument on a morphological phylogenetic analysis, but did not elaborate on either characters or relationships within the subclade they recognised, which also included *Colona* Cav., *Desplatsia* Bocq., *Mollia* Mart., *Tetralix* Griseb. and *Trichospermum*, based on molecular data and *Duboscia* Bocq. and *Vasivaea* Baill., based solely on morphological data. Their phylogenetic analysis did not support the inclusion of *Eleutherostylis* within *Grewia*, although there was not strong support for any of the relationships that they found. Jennings (2021) treated *Eleutherostylis* in a floristic account of the trees of New Guinea, but the key couplet in that work used to separate *Eleutherostylis* from *Grewia* is based only on leaf and stipule characters.

Pollen data presented by Perveen et al. (2004) do not permit one to distinguish *Eleutherostylis* from *Grewia*. The pollen grains of both are described as coarsely reticulate and 3-colporate. The pollen shape ratio (P/E) of the former (1.66) falls within the range (1.27–2.05) of the limited number of species sampled of the latter. Pollen grains of both genera, prolate with long colpi, are described as “Corchorus-type”, which can be applied to most Grewioideae, except for *Apeiba* Aubl., which Brunken and Muellner (2012) placed in Apeibeae Benth., a tribe distinct from Grewioideae. Hoorn et al. (2019), relying on the information and images provided by Perveen et al. (2004), noted that, within Malvaceae, the occurrence of prolate–subprolate bireticulate pollen with long colpi and distinct margos is restricted to Grewioideae.

*Eleutherostylis* was recently sampled with genomic-scale data for the Plant and Fungal Trees of Life Project (PAFTOL) and it was resolved as the strongly-supported sister-group to the single *Grewia* sampled (*G.flavescens* Juss.) (see Tree of Life Explorer, https://treeoflife.kew.org/tree-of-life; Baker et al. (2022)). The PAFTOL generic-exemplar approach to taxon sampling, however, did not test the monophyly of *Grewia*, which requires a more synoptic representation for that genus. The goals of our study, as part of broader phylogenetic work within Grewioideae, were to test the relationships of *Eleutherostylis* more adequately with respect to *Grewia*.

## ﻿Material and methods

**Figure 1.**
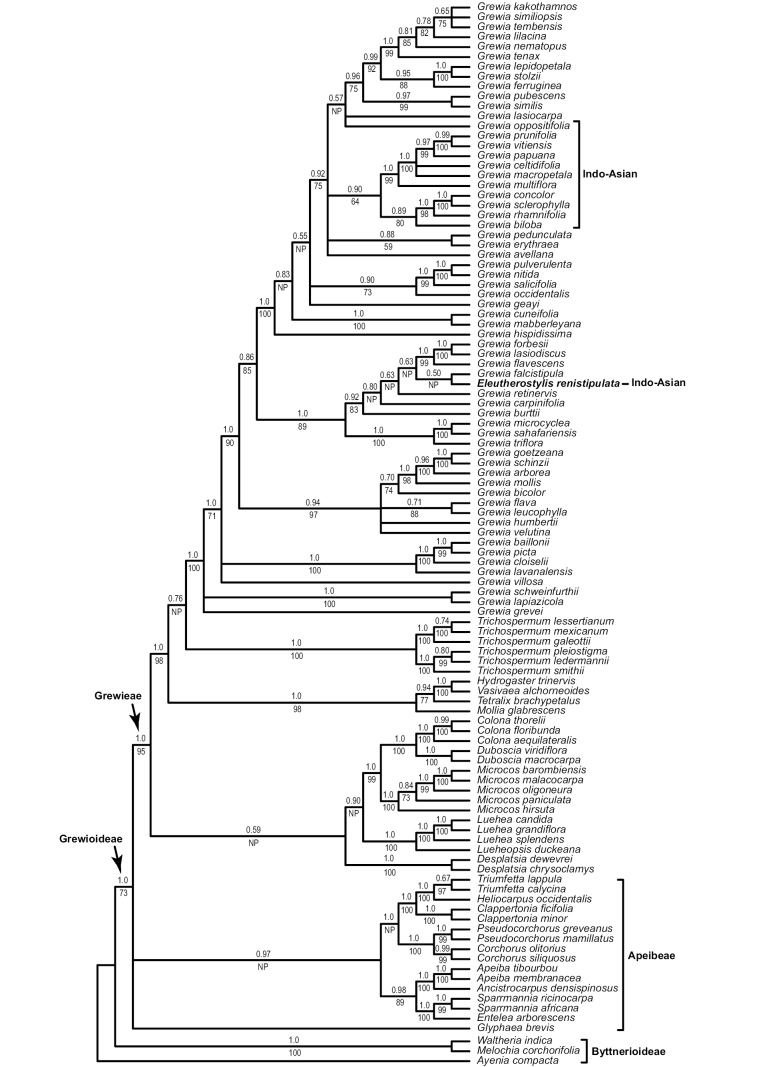
Phylogenetic relationships of *Eleutherostylis* and its Grewioideae relatives. Bayesian 50% majority-rule consensus tree, based on ITS sequences with posterior probability and IQ-TREE ML bootstrap values indicated, above and below branches, respectively. NP = an edge not present with ML.

The taxon sampling here of 107 tips was a subset of data from an ongoing broader phylogenetic study and included *Eleutherostylisrenistipulata* (*Schodde & Craven 4438*, US [01210662], the neotype designated below), along with a wide geographic sampling of *Grewia* (61 species) and 23/25 genera of Grewioideae (missing *Erinocarpus* Nimmo ex J. Graham and *Goethalsia* Pittier, which have been sampled elsewhere) recognised by Bayer and Kubitzki (2003). Outgroups included three Byttnerioideae (Malvaceae). Appendix 1 provides details of data sources, including vouchers and GenBank accession numbers. The nuclear ribosomal Internal Transcribed Spacer (ITS) region was selected for its ability to resolve the relevant taxa and recoverability from herbarium-specimen sourced DNAs. While single/few locus phylogenetic studies may appear dated in this genomics era, they remain appropriate for the scale of questions addressed here. Molecular methods for the 97 newly-generated sequences and phylogenetic analyses largely followed Dorr and Wurdack (2021) from DNeasy Plant Mini Kit (Qiagen Inc., Valencia, California, USA) extractions through to fluorescent Sanger sequencing of ITS amplification products on an ABI 3730xl DNA Analyzer (Thermo Fisher Scientific, Waltham, Massachusetts). The multiple sequence alignment (MSA) used MAFFT v.7.272 (Katoh and Standley 2013) under the G-INS-i refinement method, followed by minor manual refinements, based on a similarity criterion. Sensitivity analyses with different MAFFT optimality criteria, alignment without the divergent Byttnerioideae outgroups and exclusion sets to reduce missing data (i.e. removal of 119 MSA columns with > 50% missing data that reduced overall missing data from 16.2 to 3.85%) had little impact on the phylogenetic resolution, except in a few poorly-supported nodes. While there is sequence length variation within *Grewia*, there are few ambiguously aligned regions within our sampling for the genus. The MSA with the tested exclusion set is archived in the Dryad data repository (https://doi.org/10.5061/dryad.cnp5hqcbx). Final Maximum Likelihood (ML) analyses using all data were with IQ-TREE v.1.6.12 (Trifinopoulos et al. 2016) under GTR+F+I+G4 (selected by ModelFinder; Kalyaanamoorthy et al. (2017)) and RAxML-NG (Stamatakis 2014) as implemented on CIPRES XSEDE under GTR+I+Γ and clade support estimated by 1000 rapid bootstrap replicates. Bayesian Inference (BI) under GTR+I+Γ was performed using MrBayes v.3.2.7a (Ronquist et al. 2012) with two concurrent runs, each with four Markov chains (three cold and one heated), a 0.2 temperature coefficient and sampling every 1000 generations over 50 million generations and a conservative 25% burn-in. Topology and support value differences between the ML programmes were slight (IQ-TREE values are presented in Fig. 1); however, more pronounced were ML versus BI differences with shifts in some deeper nodes (albeit mostly poorly supported).

## ﻿Results

*Eleutherostylis* is resolved as well-nested with multiple strongly-supported nodes (Posterior probability = 1.0, ML bootstrap > 85%) within a paraphyletic *Grewia* (Fig. 1). Its sequence divergence is relatively high amongst *Grewia* species, although it does not present additional alignment problems. Relationships within *Grewia* suggest a complex biogeography, although the small taxon and gene sampling presented here limit our conclusions. The *Grewia* subclade containing *Eleutherostylis* contains only African species, while all the other Indo-Asian species (11 sampled) are far removed and mostly in a separate well-supported subclade. Relationships amongst the genera of Grewioideae and resolution as two major clades (circumscribed as tribes Apeibeae and Grewieae) largely mirror other studies (Bayer et al. 1999; Brunken and Muellner 2012; PAFTOL), except in the weakly-supported placement here of *Desplatsia* further removed from *Grewia*. *Microcos* is clearly distinct from *Grewia* and groups with *Colona* and *Duboscia*, which are Asian and African genera, respectively. *Desplatsia* and *Duboscia*, both African, are distinct from each other despite prior uncertainty (see Bayer and Kubitzki (2003)). The Neotropical and Palaeotropical taxa of *Trichospermum* group as separate sister subclades.

## ﻿Discussion

*Grewia* is a morphologically diverse and biogeographically interesting genus, with species radiations in the Paleotropics, especially in sub-arid and woodland regions of Africa with 60% of the nearly 300 species. This diversification of arid-adapted shrubby species, often with animal dispersed fleshy drupaceous fruit, is especially notable in Madagascar (ca. 65 species, mostly endemic) and Sub-Saharan Africa (124 species) (Gautier et al. 2012). The Indo-Asian (including China) diversity of *Grewia* contains ca. 73 species, with a subset of only 28 species in the Flora Malesiana Region (Peninsula Malaysia, Indonesia, New Guinea, Philippines and Borneo). The relatively isolated nature of *Eleutherostylis* is suggested by sequence divergence and its placement amongst African taxa away from the Indo-Asian clade. However, our limited taxon sampling (ca. 20% of *Grewia* species) and limited phylogenetic resolution prevent us from reliably identifying finer patterns in biogeography, except to note that the Malagasy endemics do not form a monophyletic group. While *Eleutherostylis* fits well within the broad morphological diversity of *Grewia*, it is ecologically unusual in the genus inasmuch as it is a large dioecious tree with dry fruit in lowland tropical forests. Most Malvaceae are hermaphroditic and dioecy is rare. Within Grewioideae, dioecy characterises *Hydrogaster* Kuhlm., *Tetralix*, *Vasivaea* and some *Grewia* (including *Eleutherostylis*). In addition, *Erinocarpus*, *Grewia*, *Heliocarpus* L. and *Triumfetta* L. have some species with alternative breeding systems (e.g. polygamous, gynodioecious) and sometimes unisexual (staminate, pistillate) flowers. While the stipules of *Eleutherostylis* are conspicuous because of their relatively largely size and positional dimorphism, similar large stipules occur in other species of *Grewia* (e.g. *G.falcistipula* K. Schum., an African species).

### ﻿Taxonomic summary

#### ﻿ Grewia renistipulata

Taxon classificationPlantaeMalvalesMalvaceae

(Burret) Dorr comb. nov.

18FDF13F-0843-5349-AE38-6FDFBE9BC2F2

urn:lsid:ipni.org:names:77334716-1

Fig. 2

Eleutherostylis renistipulata Burret, Notizbl. Bot. Gart. Berlin-Dahlem 9(88): 630. 1926. Type: New Guinea. Gulf District: West bank of Vailala River, ca. 3 mi south of junction with Lohiki River, ca. 18 m elev., 3 Feb 1966 (fl, fr), *R. Schodde & L.A. Craven 4438* (neotype, here designated: US [01210662]!; isoneotypes: A-2 sheets!, BH!, BO, BRI, CANB [162189.1], CANB [162190.3], CANB [162191.2], G, K [K000062278 as image!], L [L.2349560 as image!], LAE, PNH).
Grewia morotaiensis Kosterm., Reinwardtia 7(5): 444, fig. 5. 1969 (p. 444 as “*raorotaiensis*”; fig. 5 as “*morotaiensis*”). Type: Indonesia. Moluccas, Morotai Isl., Distr. Tobelo near Totodoku [sic], 30 m elev., 2 May 1949 (fr), *A.J.G.H. Kostermans & W. Tangkilisan 44* (= *bb 33752*) (lectotype, here designated: [BO-1331873 as image!]; isolectotypes: A, BM, BO [BO-1331874 as image!], K, L [L 0062741 as image!], LAE, P, PNH, SING [0054550]).

##### Additional specimens examined.

Indonesia. Moluccas, Morotai, Subdistr. Tobelo, north near Totodokoe, 30 m elev., 9 May 1949 (fl), *A.J.G.H. Kostermans & W. Tangkilisan 101* (= *bb 33795*) (A, BO [BO-1331875 as image!], K, L [L.2349554 as image!], L [L.2349555 as image!], SING). New Guinea. [Papua New Guinea]. Gulf District, Delta Divn., Baroi Riv., near Port Romilly, 26 Feb 1951, *M.F.C. Jackson 4119* (BISH, BRI, L [L.2349551 as image!], LAE). Madang District: ca. 5 km SE of Faita Village, along the Ramu River, ca. 150 m elev., 29 Jul 1955 (fl), *R.D. Hoogland 5044* (BRI, CANB [76047.1], CANB [76047.2], L [L.2349552 as image!], L [L.2349561 as image!], LAE, MEL [MEL 2370751A]!). Madang District, Gogol Base, 18 Aug 1969 (♂), *M. Kumul W. 2672* (A!, L [L.2349562 as image!]). Indonesia [Netherlands New Guinea]. Hollandia, 29 Oct 1954 (fr), *A. Brower Bw. 1611* (L [L.2349558 as image!], LAE). Sekoli Plain, Div. Hollandia, ca. 100 m elev., 25 Feb 1960, *G. Th. Iwanggin BW 9206* (BISH (×2), CANB [518317.1], L [L.2349557 as image!], WAG [WAG.1834811 as image!], WAG [WAG.1834812 as image!]). Sekoli- Plain, Div. Hollandia, ca. 100 m elev., 26 Feb 1960, *G. Th. Iwanggin BW 9222* (BISH, CANB [51833.1], L [L.2349564 as image!], LAE). Hollandia, Berap (Nimboeran), 9 Aug 1939, *Neth. Ind. For. Service bb.28959* (L [L.2349556 as image!]). W. Irian, Dozai, E. of Sukarnapura (= Hollandia), 50 m elev., 24 Aug 1966 (♂), *A.J.G.H. Kostermans & W. Soegeng 554* (CANB [339882.1], L [L.2349559 as image!]). Sekoli, O. Afd. Hollandia, 75 m elev., 26 Feb 1960, *A. Noesi BW 8147* (L [L.2349563 as image!], LAE). Tami, Hollandia, 19 Jan 1955, *F. Schram BW 1639* (CANB [51612.1], L [L.2349553 as image!], LAE).

##### Nomenclatural notes.

**Figure 2.**
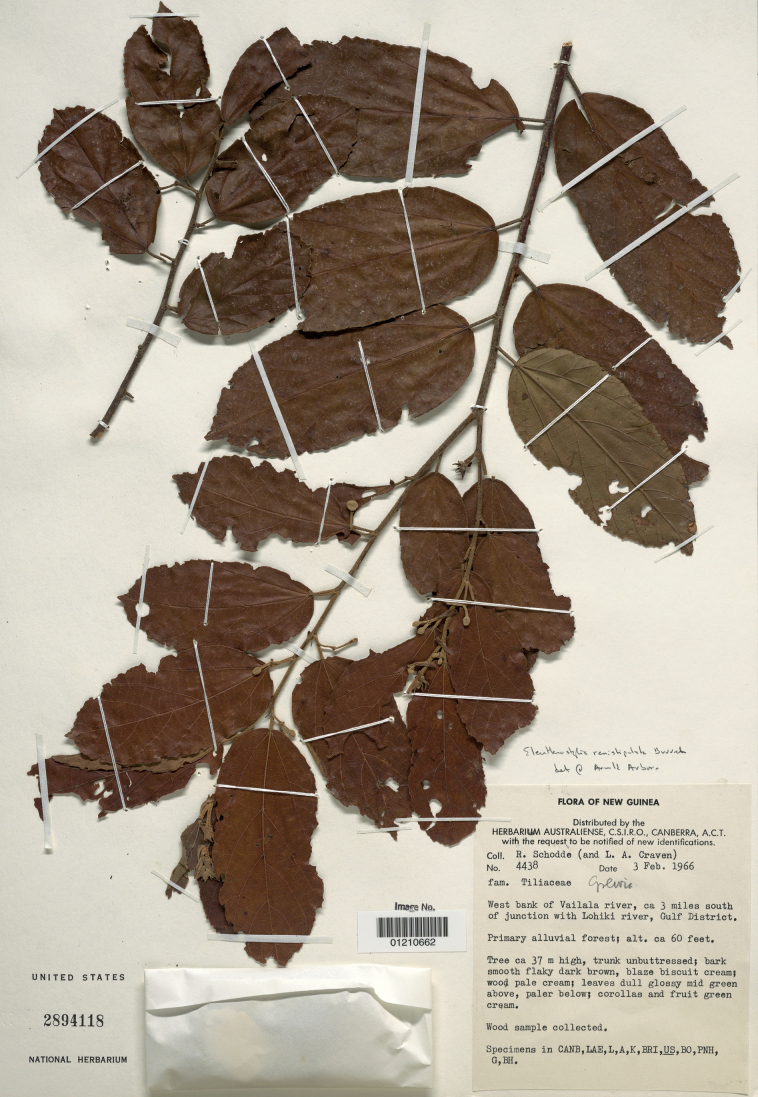
Neotype of *Eleutherostylisrenistipulata* Burret (US [01210662]).

Burret (1926) based *Eleutherostylisrenistipulata* on two collections (syntypes) made by C.L. Ledermann in Sepik, Papua New Guinea: *Ledermann 10535* with male flowers and *Ledermann 10754* with female flowers and fruit. Neither of these syntypes, which were in Berlin (B), survived World War II (Juraj Paule, pers. comm.; see also Hiepko (1987)). Steenis-Kruseman (1950, Steenis-Kruseman 1958) noted that, in addition to Ledermann collections deposited in B, duplicate material sometimes can be found in E, K, L and SING, but no duplicates of the syntypes have been located in these Herbaria. Hence, the designation here of a neotype for the name (Fig. 2).

Kostermans (1969) used two different epithets in the original description of *Grewiamorotaiensis*: “raorotaiensis” in the text and “morotaiensis” in the figure caption. These epithets have equal priority. Subsequently, Kostermans (1972) effectively made a choice between these competing epithets when he placed *G.morotaiensis* in synonymy under *Eleutherostylisrenistipulata* (see Turland et al. (2018; Art. 11.5)).

## Supplementary Material

XML Treatment for Grewia renistipulata

## ﻿Additional information

### Author ORCIDs

Laurence J. Dorr https://orcid.org/0000-0001-7157-363X

Kenneth J. Wurdack https://orcid.org/0000-0003-0010-6932

### Data availability

All of the data that support the findings of this study are available in the main text or were deposited in archives (Dryad or GenBank).

## ﻿Appendix 1

Vouchers

Sources for data used in the phylogenetic analysis of Grewioideae. Ordered as: Taxon, Voucher (new data only; bc = barcode) and GenBank number (new data begin with OR).

*Ancistrocarpusdensispinosus* Oliv., Cameroon, *X. van der Burgt et al. 936* (MO-6147053) OR934538. *Apeibamembranacea* Spruce ex Benth., Bolivia, *E. Stijfhoorn 705* (US-3266700; bc 0053551), OR934539. *Apeibatibourbou* Aubl., Bolivia, *S. Paredes 427* (US-3684178; bc 01183679), OR934540. *Ayeniacompacta* Rose, MF963978. *Clappertoniaficifolia* (Willd.) Decne., Ghana, *D. Harder et al. 3447* (MO-5030390), OR934541. *Clappertoniaminor* (Baill.) Bech., Ghana, *H. Schmidt et al. 2081* (MO-5019391), OR934542. *Colonaaequilateralis* (C.T.White & W.D.Francis) Merr. & L.M.Perry, New Guinea, *L. Craven & R. Schodde 1014* (US-3320995; bc 01317130), OR934543. *Colonafloribunda* (Kurz) Craib, KR531939 (ITS2 only). *Colonathorelii* (Gagnep.) Burret, KR531940 (ITS2 only). *Corchorusolitorius* L., KY968914. *Corchorussiliquosus* L., FJ527604. *Desplatsiachrysochlamys* (Mildbr. & Burret) Mildbr. & Burret, Central African Republic, *D. Harris & J. Fay 680* (US-3404805; bc 01210682), OR934544. *Desplatsiadewevrei* (De Wild. & T.Durand) Burret, Uganda, *ATBP 572* (MO-4935375), OR934545. *Dubosciamacrocarpa* Bocq., Gabon, *J. Reitsma & B. Reitsma 2260* (NY), OR934546. *Dubosciaviridiflora* (K.Schum.) Mildbr., Ghana, *C. Jongkind et al. 1807* (MO-4667359), OR934547. *Eleutherostylisrenistipulata* Burret, New Guinea, *R. Schodde & L. Craven 4438* (US-2894118; bc 01210662), OR934548. *Enteleaarborescens* R.Br., FR875089. *Glyphaeabrevis* (Spreng.) Monach., Ghana, *H. Schmidt et al. 1618* (MO-504354), OR934549. *Grewiaarborea* (Forssk.) Lam., Tanzania, *P. Kuchar 24804* (MO-5688296), OR934550. *Grewiaavellana* Hiern, Namibia, *S. Rugheimer et al. 2980* (US-3702461; bc 01183956), OR934551. *Grewiabaillonii* R. Vig., Madagascar, *R. Noyes et al. 1011* (MO-5054318), OR934552. *Grewiabicolor* Juss., Namibia, *L. Nanyeni 311* (US-3702456; bc 01183964), OR934553. *Grewiabiloba* G. Don, China, *D. Boufford et al. 37887* (MO-6139773), OR934554. *Grewiaburttii* Exell, Tanzania, *P. Kuchar 23193* (MO-5583151), OR934555. *Grewiacarpinifolia* Juss., Ghana, *C. Jongkind 1707* (MO-4666899), OR934556. *Grewiaceltidifolia* Juss., China, *L. Heng 8587* (MO-5320749), OR934557. *Grewiacloiselii* R.Vig., Madagascar, *T. Croat 31963* (MO-2599538), OR934558. *Grewiaconcolor* Merr., China, *N. Chun & C. Tso 44606* (US-1675394; bc 01173074), OR934559. *Grewiacuneifolia* Juss., Madagascar, *R. Gereau et al. 5733* (US-3335856; bc 00833950), OR934560. *Grewiaerythraea* Schweinf., Ethiopia, *D. Sebsebe & A. Berhanu 1996* (MO-3887915), OR934561. *Grewiafalcistipula* K.Schum., Namibia, *S. Bartsch et al. 1800* (US-3599415; bc 00814074), OR934562. *Grewiaferruginea* Hochst. ex A.Rich., Tanzania, *S. Bidgood et al. 3492* (MO-4925265), OR934563. *Grewiaflava* DC., Namibia, *S. Rugheimer et al. 2504* (US-3702462; bc 01183961), OR934564. *Grewiaflavescens* Juss., Namibia, *S. Rugheimer et al. 2669* (US-3702455; bc 01183967), OR934565. *Grewiaforbesii* Harv. ex Mast., Tanzania, *R. Gereau et al. 6624* (MO-5334011), OR934566. *Grewiageayi* R.Vig., Madagascar, *F. Chauvet 397* (MO-6332845), OR934567. *Grewiagoetzeana* K.Schum., Tanzania, *A. Ntemi Sallu 665* (MO-5753794), OR934568. *Grewiagrevei* Baill., Madagascar, *P. Phillipson 1680* (MO-3514731), OR934569. *Grewiahispidissima* Wahlert et al., Madagascar, *J. Razafitsalama & Torze 1091* (US-3547850; bc 01205465), OR934570. *Grewiahumbertii* Capuron, Madagascar, *P. Phillipson 2536* (MO-3662965), OR934571. *Grewiakakothamnos* K.Schum., Ethiopia, *C. Puff 870423-315* (MO-4010325), OR934572. *Grewialapiazicola* Capuron, Madagascar, *M. Bardot-Vaucoulon & G. Véné 1782* (MO-6152266), OR934573. *Grewialasiocarpa* E.Mey. ex Harv., South Africa, *P. Phillipson 4381* (MO-5650943), OR934574. *Grewialasiodiscus* K.Schum., Mali, *C. Duvall 91* (MO-5333223), OR934575. *Grewialavanalensis* Baill., Madagascar, *P. Phillipson 3092* (US-3398970; bc 01205481), OR934576. *Grewialepidopetala* Garcke, Tanzania, *Y. Abeid et al. 1860* (MO-4797160), OR934577. *Grewialeucophylla* Capuron, Madagascar, *P. Phillipson & S. Rabesihanaka 3143* (US-3398976; bc 01205489), OR934578. *Grewialilacina* K.Schum., Tanzania, *V. Simon & Y.S. Abeid 69* (MO-4902872), OR934579. *Grewiamabberleyana* Phillipson et al., Madagascar, *G. Schatz 3356* (MO-6719378), OR934580. *Grewiamacropetala* Burret, China, *Qin Hai-ning et al. 2362* (MO-5746218), OR934581. *Grewiamicrocyclea* (Burret) Capuron & Mabb., Madagascar, *P. Phillipson 2908* (US-3404759; bc 01207518), OR934582. *Grewiamollis* Juss., Tanzania, *F. Mawi et al. 182* (MO-5731705), OR934583. *Grewiamultiflora* Juss., Burma, *R. Belcher* [*US Typhus Comm.*] *671* (US-2213169; bc 00685866), OR934584. *Grewianematopus* K.Schum., Kenya, *Y. Harvey et al. 60* (MO-5779207), OR934585. *Grewianitida* Juss., Madagascar, *G. McPherson et al. 14761* (MO-3771574), OR934586. *Grewiaoccidentalis* L., South Africa, *G. Germishuizen 952* (US-3022273; bc 01207559), OR934587. *Grewiaoppositifolia* Buch.-Ham. ex DC., India, *W. Koeltz 4446* (US-1608157; bc 01173134), OR934588. *Grewiapapuana* Burret, Australia, *G. Batianoff 900146* (MO-4341230, OR934589. *Grewiapedunculata* K.Schum., Mozambique, *W. Luke et al. 9907* (MO-5761031), OR934590. *Grewiapicta* Baill., Comoros, *J.-N. Labat 3198* (MO-6113815), OR934591. *Grewiaprunifolia* A.Gray, Fiji, *A. Smith 7910* (US-2190725; bc 00813934), OR934592. *Grewiapubescens* P.Beauv., Guinea, *C. Jongkind 8345* (MO-6185625), OR934593. *Grewiapulverulenta* R.Vig., Madagascar, *G. McPherson & N. Dumetz 14317* (MO-3771579), OR934594. *Grewiaretinervis* Burret, Namibia, *L. Hoffman 568* (US-3702466; bc 01183958), OR934595. *Grewiarhamnifolia* B.Heyne ex Roth, Sri Lanka, *A. Robyns 7209* (US-2660528; bc 00813779), OR934596. *Grewiasahafariensis* Capuron & Mabb., Madagascar, *R. Capuron 23036-SF* (MO-6354013), OR934597. *Grewiasalicifolia* Schinz, Seychelles (Aldabra), *S. Renvoize 1346* (US-2779483; bc 01207633), OR934598. *Grewiaschinzii* K.Schum., Botswana, *P. Smith 4360* (MO-5988288), OR934599. *Grewiaschweinfurthii* Burret, Ethiopia, *D. Sebsebe & B. Tamirat 2349* (MO-3855548), OR934600. *Grewiasclerophylla* Roxb. ex G.Don, Nepal, *R. Troth 704* (US-2826357; bc 01173161), OR934601. *Grewiasimiliopsis* C.Whitehouse, Tanzania, *P. Kuchar 24245* (MO-5700280), OR934602. *Grewiasimilis* K.Schum., Tanzania, *L. Ellemann 645* (MO-4859391), OR934603. *Grewiastolzii* Ulbr., Tanzania, *J. Lovett & C. Kayombo 4389* (US-3362978; bc 01207665), OR934604. *Grewiatembensis* Fresen., Saudi Arabia, *T. Miyazaki 990915R5* (MO-6334153), OR934605. *Grewiatenax* (Forssk.) Fiori, Namibia, *H. Kolberg et al. 1215* (US-3702464; bc 01183955), OR934606. *Grewiatriflora* (Bojer) Walp., Madagascar, *F. Ratovoson et al. 916* (MO-6034468; bc MO-1059913), OR934607. *Grewiavelutina* (Forssk.) Lam., Saudi Arabia, *T. Miyazaki 990905R1* (MO-6334157), OR934608. *Grewiavillosa* Willd., Saudi Arabia, *J. Lavranos & I. Collenette 20449* (MO-2952050), OR934609. *Grewiavitiensis* Turrill, Fiji, *O. Degener 15242* (US-1944019; bc 00980012), OR934610. *Heliocarpusoccidentalis* Rose, Mexico, *R. McVaugh 20863* (US-2151641; bc 00520160), OR934611. *Hydrogastertrinervis* Kuhlm., Brazil, *J. Paixao et al. 554* (NY-884904), OR934612. *Lueheacandida* (DC.) Mart., Mexico, *J. Amith 1843* (US-3635420; bc 00976739), OR934613. *Lueheagrandiflora* Mart., Brazil, *B. Amorim et al. 523* (US-3645266; bc 01183510), OR934614. *Lueheasplendens* Rusby, Bolivia, *R. López & M. Capra 825* (US-3670168; bc 01183716), OR934615. *Lueheopsisduckeana* Burret, Brazil, *G. Árboez et al. 4305* (US-3419295; bc 00727607), OR934616. *Melochiacorchorifolia* L., MH768329. *Microcosbarombiensis* (K.Schum.) Cheek, Cameroon, *X. van der Burgt & J. Amambo 379* (MO-5779211), OR934617. *Microcoshirsuta* (Korth.) Burret, Indonesia, *C. Peters 1025* (US-3290456; bc 01208471), OR934618. *Microcosmalacocarpa* (Mast.) Burret, Ghana, *H. Schmidt et al. 2022* (MO-5006532), OR934619. *Microcosoligoneura* (Sprague) Burret, Gabon, *J. Boussengui-Nongo 281* (MO-6311890), OR934620. *Microcospaniculata* L., KP092996. *Molliaglabrescens* Benth., Guyana, *K. Redden et al. 6241* (US-3572625; bc 01074205), OR934621. *Pseudocorchorusgreveanus* (Baill.) Capuron, Madagascar, *J. & M. Peltier 5196* (MO-6356087), OR934622. *Pseudocorchorusmamillatus* Capuron, Madagascar, *R. Bolliger 337* (MO-6612809), OR934623. *Sparrmanniaafricana* L.f., South Africa, *R. Brand 228* (MO-5652084), OR934624. *Sparrmanniaricinocarpa* (Eckl. & Zeyh.) Kuntze, FR875088. *Tetralixbrachypetalus* Griseb., Cuba, *R. Berazaín et al. [JBNC] 71840* (NY), OR934625. *Trichospermumgaleottii* (Turcz.) Kosterm., Mexico, *P. Acevedo-Rdgz. et al. 16062* (US-3697233, bc: 01343493), OR934626. *Trichospermumledermannii* Burret, Palau (Caroline Islands), *F. Fosberg 50600* (US-3408397; bc 01165395), OR934627. *Trichospermumlessertianum* (Hochr.) Dorr, Mexico, *G. Martínez Calderón 1651* (US-3571535; bc 00976860), OR934628. *Trichospermummexicanum* (DC.) Baill., Mexico, *J. Amith 1817* (US-3635429; bc: 00976741), OR934629. *Trichospermumpleiostigma* (F. Muell.) Kosterm., Papua New Guinea, *W. Takeuchi 4559* (US-3306729 [sic] US-3716430 bc: 04214468), OR934630. *Trichospermumsmithii* Kosterm., Fiji, *J. Gressitt 2499* (US-2210382; bc: 00977330), OR934631. *Triumfettacalycina* Turcz., Peru, *W. Galiano et al. 4835* (US-3534756; bc 00755457), OR934632. *Triumfettalappula* L., Panama, *P. Peterson & C. Annable 7242* (US-3114850; bc 00501649), OR934633. *Vasivaeaalchorneoides* Baill., Guyana, *M. Jansen-Jacobs et al. 34* (US-3070508; bc 00513683), OR934634. *Waltheriaindica* L., MH768330.
